# Equality and Freedom as Uncertainty in Groups

**DOI:** 10.3390/e23111384

**Published:** 2021-10-22

**Authors:** Jesse Hoey

**Affiliations:** David R. Cheriton School of Computer Science, Univeristy of Waterloo, Waterloo, ON N2L 3G1, Canada; jhoey@cs.uwaterloo.ca

**Keywords:** free energy, uncertainty, POMDP, active inference, emotion, affect control theory, sociology

## Abstract

In this paper, I investigate a connection between a common characterisation of freedom and how uncertainty is managed in a Bayesian hierarchical model. To do this, I consider a distributed factorization of a group’s optimization of free energy, in which each agent is attempting to align with the group and with its own model. I show how this can lead to equilibria for groups, defined by the capacity of the model being used, essentially how many different datasets it can handle. In particular, I show that there is a “sweet spot” in the capacity of a normal model in each agent’s decentralized optimization, and that this “sweet spot” corresponds to minimal free energy for the group. At the sweet spot, an agent can predict what the group will do and the group is not surprised by the agent. However, there is an asymmetry. A higher capacity model for an agent makes it harder for the individual to learn, as there are more parameters. Simultaneously, a higher capacity model for the group, implemented as a higher capacity model for each member agent, makes it easier for a group to integrate a new member. To optimize for a group of agents then requires one to make a trade-off in capacity, as each individual agent seeks to decrease capacity, but there is pressure from the group to increase capacity of all members. This pressure exists because as individual agent’s capacities are reduced, so too are their abilities to model other agents, and thereby to establish pro-social behavioural patterns. I then consider a basic two-level (dual process) Bayesian model of social reasoning and a set of three parameters of capacity that are required to implement such a model. Considering these three capacities as dependent elements in a free energy minimization for a group leads to a “sweet surface” in a three-dimensional space defining the triplet of parameters that each agent must use should they hope to minimize free energy as a group. Finally, I relate these three parameters to three notions of freedom and equality in human social organization, and postulate a correspondence between freedom and model capacity. That is, models with higher capacity, have more freedom as they can interact with more datasets.

## 1. Introduction

My primary objective in this paper is to propose a computational model which may give insights into the deep level of cooperation observed in human groups. While much of economics and artificial intelligence have focussed on arbitrarily modifying a utility function (e.g., with incentives for “fairness” [1], “influence” [2], “envy” [3], or “altruism” [4,5]; see my review in [6]), this still requires an agent to solve an intractable social coordination problem:

*“[…] a rational-choice model of collective action, in which individuals calculate that they will be better off cooperating with one another, vastly understates the degree of social cooperation that exists in human societies and misunderstands the motives that underlie it*.

([7], p. 439)

One possible explanation for how humans achieve this high level of cooperation is by figuring out who predicts, explains and generates what in a group, or how the epistemic labour is divided. While each individual can come up with some reasonable predictions, many of these will have flaws that can be uncovered by an opposing viewpoint, or will be invalidated by data. However, each individual will be overtaxed if asked to come up with, and compare, *every possible* solution. Therefore, the group will be more efficient if they spread out, each member trying to push a different viewpoint. The more viewpoints, the better. The search through epistemic space by the group to locate a position of minimal free energy will be handled by fanning out, but not so far apart that they cease to be a coherent group, as security is compromised. Intelligence, innovation, and learning therefore lie in diversity [8].

In this paper, I propose a computational model of this cooperation mechanism based on the management of uncertainty in a hierarchical Bayesian model. I show how agents that manage their uncertainty in the same way will have a “sweet spot” at which they best fit the group and the group best fits them. In order to make this more concrete, I use a two-level Bayesian model in which the “higher” level in the model represents *shared dynamic models of state and action based on cognitive social emotions.* These social emotional models are based on processual symbolic interactionist ideas arising in sociology [9]. I argue that these shared dynamics are useful to help a *group* of people find a free energy minimum, as they would be expected to do under the free energy principle (FEP) [10]. At this minimum, they are coordinated to the best degree possible: each individual fits the group and the group fits each individual as well as possible given variations in a huge variety of attributes across different group members. The inclusion of *action* (really, *policy*, or *strategy*) in these shared dynamic models means that not only is this alignment across states of belief, but it is also across *intents*, or what group members are planning to do in in the future. According to FEP, at equilibrium, each agent suffers the least surprise in its social interactions *with its own group* (which may have negative externality of an increase in free energy *outside the group*). In order to keep the free energy of each individual and that of the entire group to a minimum, a trade-off must be made, which is the primary subject of this paper.

I aim to show, in an upwards reduction, that a mathematical trade-off exists in the structure of multi-agent system cooperative action problems. This trade-off is conjectured in this paper to be externalized by people in their social econiches, in particular in their beliefs about equality and freedom. I will start this by looking at a single-dimensional space, and show that by factoring a free energy formulation of beliefs into two parts, an information asymmetry arises between individual agents (who act as “principals” here) and a group of agents (who act as “agent” here). The abstractions created in the mind, such as the conscious experience of language, necessarily discard information. A family of objects given a certain label must contain more information, or have higher capacity, than any individual object in the family. This creates a tension between top-down prediction, which is individually driven, and bottom up evidence, which is driven by a group. The individual favours simple models, as they require less cognitive effort, but these come with increased information hiding by the group. The group, on the other hand, favours more complex models, as these are more flexible to changing inputs (they can model more datasets). Therefore, a balance is sought in the complexity (or *“capacity”*) of the model selected.

I also conjecture that diversity in a group can be translated into model capacity in each agent’s mind because of the good regulator theorem: every operational system has to be a model of its environment [11], which may be social (may include other agents). Thus, each agent is both defined by, and defines, the group it interacts with. If agents are defined by a group, yet agents must be diverse, this uncovers the trade-off. I make the simplifying assumption in this paper of a single group, while in practice, people are simultaneously in groups that span multiple scales of organization. Sitting with your friend in class is such a situation, as you are in two groups: friends and classmates.

There are two ways of organizing a social group, and of organizing each agent’s model: precise and homogeneous, or uncertain and diverse. These two ways lead to solutions that are secure and static or insecure and innovative, respectively. Finally, I will claim that these methods correspond to one possible definition of equality and freedom, also respectively. They cannot be achieved at the same time, yet each has its advantages. The argument that the social structure is reflected in the human mind, and vice versa, lies at the heart of this conjecture. Beyond the good regulator theorem, the “social construction of reality” is precisely the idea that social structures (reality) are constructed in the mind, and vice versa [12]. Diversity in a society for example, leads to more liberal political structures emerging. It is precisely the increased uncertainty in each agent’s mind that leads to this conclusion.

How will an agent choose between these two organizational methods? Being very certain about things is good because it allows decisions to be made, as an agent’s certainty in something needs to be raised above a threshold for action. Being uncertain about things is also good because it allows an agent to be flexible towards changing situations and new and different people. However, these extreme values are difficult to sustain a social order over. The reason is that, in a state of perfect freedom, no cooperation is possible: there’s just too much diversity. Similarly, a state of perfect equality will not succeed because everyone has to be identical. In this case, while everyone is very secure, the system has become very brittle to intrusions or exogenous changes, and remains stagnant (non-innovative).

**Figure 1:**
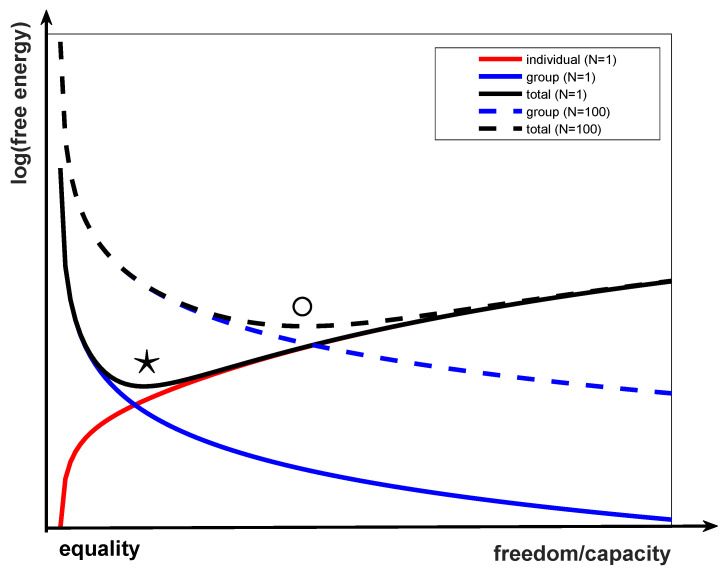
Free energy as a function of model capacity and/or freedom/equality. I plot the log(free energy) for clarity only. An arbitrary scaling of log(free energy) can be assumed, so only relative size matters. Lower free energy is a preferable situation, but the energy created by the group trying to match the individual (blue line) is balanced by that created by the individual trying to match the group (red line) to give an overall free energy which has a “sweet spot” (minimum) at ★. The black line shows the free energy if these are traded off equally. The dashed lines show the situation in which the group is much less flexible because it is larger (N = 100 times larger than the N = 1 group). Then, the “group” component of the free energy (individual stays fixed, group attempts to match, blue dashed line) is much higher, since the group’s capacity is increased, and so plays a bigger role in the resulting free energy (black dashed line), and shifts the “sweet spot” outwards towards more freedom at ∘.

I can plot a curve showing this trade-off by examining the free energy of the entire group, which splits into two terms. Figure 1 shows these two terms on a graph of the log(free energy) vs. this notion of equality and freedom I have explained in the previous paragraph. That is, to the left are systems where all group members are similar, so each individual has a minimal free energy (red curve), as it is really easy to predict everyone else since they are identical to everyone else, but the group’s free energy (blue curve) is maximal, because they are inflexible to exogenous events. To the right are systems where all group members are diverse, which has minimal free energy for the group (blue curve), because they can manipulate the division of epistemic labour, but maximal free energy for the individual, because a more complex model is required. There is therefore a sweet spot in the sum of these free energies (black curve), shown with a star in Figure 1, that trades these two off optimally in the sense that each agent is able to accurately model the group and the group is able to accurately model each agent. At this sweet spot, agent and group share a model and are best able to predict and act cooperatively in the future world. The group as a whole is functioning according to the free energy principle. Smaller free energy configurations are better because they ensure there is less “surprise” for the group and its members. It is nevertheless true that any particular group may look very different to any other group, and so this sweet spot is only universal in an information theoretic way. The precise circumstances surrounding any group may result in a different, or non-decreasing, optimization.

This sweet spot is the configuration of both agent and group such that the free energy of the group is minimized, and it arises from the group leveraging the second law of thermodynamics for its own benefit by amassing orderly states (information) at the expense of externalities [13]. In some sense, the group has transferred as much energy as possible into maintaining a state of low entropy, that is, a state of as much order as is possible given the various circumstances surrounding the group. The group and the individual are aligned in this case, and the heightened collective consciousness, regardless of how it is implemented, allows individuals to be more free to think, be and do [14].

In the next section, I derive the curves in Figure 1 for a one-dimensional parameter space. I then generalize to three dimensions, by noting that three different (sets of) parameters are needed to implement a two-level (hierarchical or deep) Bayesian model. The minimum free energy, however, requires the “participation” of all three sets as a change by any one that increases free energy will have to be offset by a change in some other set. Thus, in three dimensions, the “sweet spot” is really a “sweet surface.” The shape of this surface can be derived based on further assumptions covered in Section 2.6. Then, in Section 3, I discuss freedom and equality, and present a view of these quantities as being three dimensional and ternary, derived from social and political theorizing. Finally, in Section 4, I conjecture that the three dimensions of freedom and equality correspond to three settings of parameters in a two-level Bayesian model embedded in a multi-agent system in which agents do not have to be decision theoretically rational, but do have the capability to learn.

## 2. Free Energy

### 2.1. One-Dimensional Derivation

**Figure 2:**
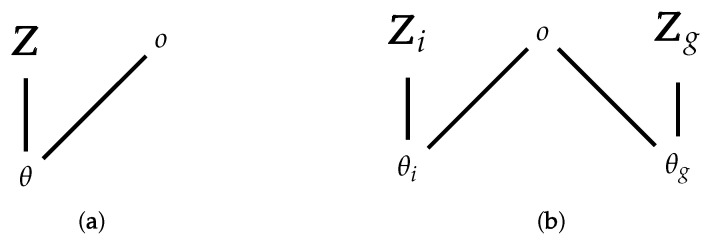
Simplified factor graph (Bayesian network) representing (**a**) a group with latent variables Z; and (**b**) the group without agent *i* with state $Z_{g}$, and one representing agent *i* with state $Z_{i}$.

I now derive the curves in Figure 1 from free energy principles. I will start with the free energy of the *whole group* of N agents. I will denote the $i^{th}$ agent’s parameters as $\theta_{i}$ and the parameters of the whole group as $\theta \equiv \{\theta_{1},\ldots ,\theta_{N}\}$. Thus, the task of the group at time *t*, given data as observations ($D={\left\{o\right\}}_{t}=\left\{o_{1},o_{2},\ldots ,o_{t}\right\}$), is to compute

(1) $$P\left(\theta |D,H\right)=\frac{P(D|\theta ,H)P(\theta |H)}{P(D|H)},$$

where H is the hypothesis space (the modeling space as defined by a Bayesian hierarchical model, for example). The graphical model for these agents is shown in Figure 2a, with a single latent variable Z for the entire group. The difficult part here is the evaluation of P(D|H) since it involves a summation over all values of θ. Further, each of these terms involves sums over the hidden variables Z.

The variational free energy, $\tilde{F}$, can be written as

(2) $$\tilde{F}=\int d\theta Q\left(\theta \right)\log\frac{Q(\theta )}{P(D,\theta )}.$$

When the approximating distribution *Q* is chosen such that $\tilde{F}$ is minimized, then the minimum of this $\tilde{F}$ as θ is varied is obtained when θ is the parameters of the best predictor function for this domain and agent combination [15]. The minimization process may be approximated by choosing *Q* for some fixed (current best-guess) θ, and then optimizing θ with respect to that “discovered” *Q*, and repeating this process until convergence to a local minimum, as in the expectation-maximization algorithm. By choosing the Q function appropriately, a minimization over model parameters is possible, and this minimization will not leave the parameters any worse off as far as relationship (fit) with the data goes. In many cases, *Q* may be determined from the data, but in some it may only be possible over some parameterized subset of the space of *Q*. For example, *Q* can be factored into parts corresponding to each parameter, and then each such factored *Q* can be minimized analytically one at a time, while keeping the others constant.

In order to move beyond the group to each individual agent, I will split the group into two parts. One singleton set contains the $i^{th}$ agent, with parameters $\theta_{i}$ and latent variables $Z_{i}$, and the other set contains N−1 agents {1,…,N}\i, with parameters $\theta_{g}$ and latent variables $Z_{g}$. I will consider this second set of agents as a single agent in what follows, and the network model looks as in Figure 2b. Equivalently, I assume each agent in the second set (the group) to be identical and act simultaneously and equally. In what follows, I will assume this group is *homogeneous and undifferentiated* in their overall parameter settings (which means they still may be using heterogeneous models), such that the group can be treated as an individual. At this point, I encourage the reader to think of this as a *dyadic* interaction, but it can also be viewed as an agent-group interaction, or even a group-group interaction. The role of this single “group” agent is, in fact, taken by a single individual from the group at any one time, but the statistics of interaction of the agent in question with the *whole* group is what matters. I am assuming here that this variation is sufficiently small, but in real human groups, I imagine it will be quite large.

I will now assume that the variational distribution for the group, *Q*, from the perspective of any agent *i*, can be factored into a piece for the agent, $Q_{i}\left(\theta_{i}\right)$ and a piece for the group, $Q_{g}\left(\theta_{g}\right)$, such that $Q\left(\theta \right)=Q_{i}\left(\theta_{i}\right)Q_{g}\left(\theta_{g}\right)$. As explained above, a variational solution will normally require some kind of iterative updating scheme like the expectation-maximization algorithm, which operates by optimising one parameter at a time, while holding the others fixed. This kind of iterative solution is achieved by factorizing the group into individuals optimizing their own *Q* functions, based on everyone else’s *Q* functions, assuming they are fixed. For the entire group I am considering, I am assuming that each agent can separately and independently minimize some part of the variational free energy. However, the minimization is actually performed by the whole group at the same time.

If each agent attempts to perform this maximization separately, the resulting joint effort will result in a group pressure on each individual that reciprocates the pressure of the individual on the group, although magnified by the concentration of it. What this implies is that each agent in a group, in attempting to manage its social network, will tend towards solutions that combine the agent’s own free energy, with the agent’s contribution to the free energy of the groups in which it its nested (here I consider only one level of nesting). This means I can write

(3) $$\tilde{F}=\int d\theta Q_{i}\left(\theta_{i}\right)Q_{g}\left(\theta_{g}\right)\log\frac{Q_{i}\left(\theta_{i}\right)Q_{g}\left(\theta_{g}\right)}{P_{i}\left(D|\theta_{i}\right)P_{g}\left(D|\theta_{g}\right)}.$$

Consider *D*, the total data “generated” (including actions performed) by the agent and group. I will break this into three non-overlapping sets, $D\equiv \{D_{o},D_{i},D_{g}\}$, where $D_{i}$ is the data generated during the interaction by the agent, *i*, while $D_{g}$ is the data generated by the group, *g*, and $D_{o}$ is the data generated by both simultaneously (or neither). For example, such data may be spoken/written language, or facial expressions and gestures, some of which are normally only be jointly expressed (like sharing a hug). Such data may also include physical artifacts in a shared space. The goal of the optimization is to get $D_{i}$ to be interpretable by the group, to get $D_{g}$ to be interpretable by the agent, and to get $D_{o}$ to be interpretable by both.

The denominator in Equation (3) is $P(D,\theta_{i},\theta_{g})$, but since $D_{i}$ is being generated by *i*, and assuming $D_{0}=⌀$, and constant priors $P(\theta_{i})$ and $P(\theta_{g})$, this is $P_{i}\left(D_{i}D_{g}|\theta_{i}\right)P_{g}\left(D_{g}D_{i}|\theta_{g}\right)$ (it is a “noisy or” or “mixture of experts” model) where $P_{i}$ and $P_{g}$ are the probabilistic models of the individual and of the group. Looking a little further, we note that the optimization in Equation (3) will favor $P_{i}$ and $P_{g}$ distributions with larger capacity, but that such a larger capacity $P_{i}$ requires a more difficult optimization by *i*, but a simpler optimization for the group *g*. To see why, consider this exemplar based solution. Consider that for *g* to model what *i* does, it suffices to have one member of *g* who is very similar to *i*. If using a Monte-Carlo (sample-based) solver, this model’s predictive samples would take most of the weight in the posterior distribution. The more diverse group with have larger capacity overall and will therefore be more likely to easily assimilate *i*. However, larger capacity agents work in the opposite way. For *i* to model what *g* does, it requires *i* to have a model for *every member of g*, or at least a sufficient abstraction (learned from) of all data from all group members. Should *i* not be able to do this, his free energy will increase very rapidly, as he struggles to figure out how everyone works. Individuals aim for the stability of homogeneity, while the group aims for the disorder of innovation. It is this asymmetry that is the primary focus of this paper. In the discussion, I will further elaborate on the connections between this and social and political freedom.

Agent and group will both be updating their models, $\theta_{i}$ and $\theta_{g}$, respectively, during the interaction. I will therefore simplify by assuming that each agent generates “its” data, then observes $D_{i}$ and $D_{g}$, and then generates the shared data $D_{0}$. Then I can factor

(4) $$\begin{array}{ll} P_{i}\left(D|\theta_{i}\right) & =\int_{\theta_{i}^{\prime }}P_{i}\left(D_{o},D_{i},D_{g},\theta_{i}^{\prime }|\theta_{i}\right)\\ & =\int_{\theta_{i}^{\prime }}P_{i}\left(D_{o}|D_{i},D_{g},\theta_{i}^{\prime },\theta_{i}\right)P_{i}\left(\theta_{i}^{\prime }|D_{g},D_{i},\theta_{i}\right)P_{i}\left(D_{i}|D_{g},\theta_{i}\right)P\left(D_{g}|\theta_{i}\right)\\ & =\int_{\theta_{i}^{\prime }}P_{i}\left(D_{o}|\theta_{i}^{\prime }\right)P_{i}\left(\theta_{i}^{\prime }|D_{g},D_{i},\theta_{i}\right)P_{i}\left(D_{i}|\theta_{i}\right)P_{i}\left(D_{g}|\theta_{i}\right), \end{array}$$

and

(5) $$\begin{array}{ll} P_{g}\left(D|\theta_{g}\right) & =\int_{\theta_{g}^{\prime }}P_{g}\left(D_{o},D_{i},D_{g},\theta_{g}^{\prime }|\theta_{g}\right)\\ \; & =\int_{\theta_{g}^{\prime }}P_{g}\left(D_{o}|\theta_{g}^{\prime }\right)P_{g}\left(\theta_{g}^{\prime }|D_{i},D_{g},\theta_{g}\right)P_{g}\left(D_{g}|\theta_{g}\right)P_{g}\left(D_{i}|\theta_{g}\right), \end{array}$$

where I have assumed that $D_{o}$ is generated from updated models in agent $\theta_{i}^{\prime }$ and group $\theta_{g}^{\prime }$ after seeing $D_{i}$ and $D_{g}$. Further, I have assumed each agent computes its own P(D|θ) without considering the other’s data. That is, $P_{i}\left(D_{i}|D_{g},\theta_{i}\right)=P_{i}\left(D_{i}|\theta_{i}\right)$ and $P_{g}\left(D_{i}|D_{g},\theta_{g}\right)=P_{g}\left(D_{i}|\theta_{g}\right)$. Putting Equations (4) and (5) into (3), and rearranging terms, I obtain:

(6) $$\begin{array}{ll} \tilde{F}= & \int Q_{i}\left(\theta_{i}\right)Q_{g}\left(\theta_{g}\right)\log\frac{Q_{i}\left(\theta_{i}\right)}{P_{i}\left(D_{i}|\theta_{i}\right)\int_{\theta_{i}^{\prime }}P_{i}\left(D_{o}|\theta_{i}^{\prime }\right)P_{i}\left(\theta_{i}^{\prime }|D_{g},D_{i},\theta_{i}\right)}\\ \; & +\int Q_{i}\left(\theta_{i}\right)Q_{g}\left(\theta_{g}\right)\log\frac{Q_{g}\left(\theta_{g}\right)}{P_{i}\left(D_{g}|\theta_{i}\right)P_{g}\left(D_{i}|\theta_{g}\right)P_{g}\left(D_{g}|\theta_{g}\right)\int_{\theta_{g}^{\prime }}P_{g}\left(D_{o}|\theta_{g}^{\prime }\right)P_{g}\left(\theta_{g}^{\prime }|D_{i},D_{g},\theta_{g}\right)}. \end{array}$$

Now I will evaluate this free energy at a fixed point where $\theta_{g}^{\prime }=\theta_{g}$ and $\theta_{i}^{\prime }=\theta_{i}$, and in the particular case where $D_{o}=⌀$, ergo, the group and individual are at equilibrium and do not jointly generate data. This means neither agent nor group changes parameters based on the other’s data. However, at equilibrium, it allows me to compute the integrals in closed form. Thus, in Equation (6), I can set $Q_{g}=P_{g}\left(D_{g}|\theta_{g}\right)$ and set the integrals over $\theta_{i}^{\prime }$ and $\theta_{g}^{\prime }$ in the denominators to identity (since one term picks out $\theta^{\prime }=\theta$, and the other is $P(D_{o}=⌀|\theta )=1$ they pick out the equilibrium point, which is the starting point).

With these assumptions in hand, I can rewrite Equation (6) as:

(7) $$\begin{array}{cc} \tilde{F}=\int_{\theta_{g}}Q_{g}\left(\theta_{g}\right) & \int_{\theta_{i}}Q_{i}\left(\theta_{i}\right)\log\frac{Q_{i}\left(\theta_{i}\right)}{P_{i}\left(D_{i}|\theta_{i}\right)}\\ \; & -\int_{\theta_{i}}Q_{i}\left(\theta_{i}\right)\int_{\theta_{g}}Q_{g}\left(\theta_{g}\right)\log P_{i}\left(D_{g}|\theta_{i}\right)P_{g}\left(D_{i}|\theta_{g}\right). \end{array}$$

The first term is the usual free energy for the agent, averaged over models of the group. However, assuming the group is stationary, then the free energy of the agent then resolves to its own free energy, which can be computed. The second term is the joint probability that agent *i* will be able to generate data $D_{i}$ that are interpretable by the group, and that the group will be able to generate data $D_{g}$ that are interpretable by the agent. This is taken in expectation over both models of agent and group parameters, $Q_{i}$ and $Q_{g}$.

Note the symmetry in Equation (7), in which the dispersion of $\theta_{i}$ can be large if the dispersion of $\theta_{g}$ is small, and vice-versa, but both cannot be large or small at the same time. In fact, this symmetry is quite curious because it states that individuals operating in the first regime will be well suited to interact with individuals operating in the second. That is, although they are doing things differently, they in fact are complementary. There is a trade-off between the capacity of these parameters with insufficient density if the two are large, difficulty finding the other if the two are small, leaving the agents to find trade-offs in the middle. The exact location of this trade-off is then something that must be negotiated. It also determines the sets $D_{o},D_{i},D_{g}$ defined above, since, e.g., if the dispersion of $\theta_{g}$ is small, most of the data will be generated by the group, so $D_{o}=D_{g}$. If the dispersion of $\theta_{g}$ is large, the dispersion of $\theta_{i}$ is small, and so $D_{o}=D_{i}$.

Focusing on the second term in Equation (7) only, I can expand out the logarithm and get two terms

$$-\int_{\theta_{i}}Q_{i}\left(\theta_{i}\right)\int_{\theta_{g}}Q_{g}\left(\theta_{g}\right)\log P_{i}\left(D_{g}|\theta_{i}\right)-\int_{\theta_{g}}Q_{g}\left(\theta_{g}\right)\int_{\theta_{i}}Q_{i}\left(\theta_{i}\right)\log P_{g}\left(D_{i}|\theta_{g}\right),$$

which I can optimize separately. Holding the agent fixed at $\theta_{i}^{*}$ and optimizing $\theta_{g}$ in the first, and holding the group fixed at $\theta_{g}^{*}$ and optimising $\theta_{i}$ in the second, then, this is

$$-\int_{\theta_{g}}Q_{g}\left(\theta_{g}\right)\log P_{i}\left(D_{g}|\theta_{i}^{*}\right)-\int_{\theta_{i}}Q_{i}\left(\theta_{i}\right)\log P_{g}\left(D_{i}|\theta_{g}^{*}\right).$$

Now, $D_{g}=\left\{d_{g0},d_{g1},\ldots ,d_{gN}\right\}$ and $D_{i}=\left\{d_{i0},d_{i1},\ldots ,d_{iN}\right\}$, which means, assuming all the data are independently and identically distributed given each model, we can write

$$-\sum_{j}\left[\int_{\theta_{g}}Q_{g}\left(\theta_{g}\right)\log P_{i}\left(D_{gj}|\theta_{i}^{*}\right)+\int_{\theta_{i}}Q_{i}\left(\theta_{i}\right)\log P_{g}\left(D_{ij}|\theta_{g}^{*}\right)\right].$$

I will assume that $Q_{g}$ is a “hat” function which has constant probability over $\left[\mu_{g}^{*}-\sigma_{g}^{*},\mu_{g}^{*}+\sigma_{g}^{*}\right]$ (so $\theta_{g}^{*}=\left\{\mu_{g}^{*},\sigma_{g}^{*}\right\}$). Similarly for $Q_{i}$: replace all *g* subscripts with *i*. Next, I assume that $P_{i}$ and $P_{g}$ are normal distributions with parameters $\theta_{i}=\left\{\mu_{i},\sigma_{i}\right\}$ and $\theta_{g}=\left\{\mu_{g},\sigma_{g}\right\}$. The assumption of normality for $P_{i}$ and $P_{g}$ is only to ease exposition here. In fact, these distributions are more likely to be scalable, that is, operating similarly at very different scales (non-Gaussian). With these assumptions in place, the integrals can be done analytically to yield, for each data point, a contribution to the overall free energy of (note the extra negative sign that came from the log(Normal) distributions):

(8) $$\frac{{\left(\mu_{g}+\sigma_{g}-\mu_{i}^{*}\right)}^{3}}{3\sigma_{i}^{*2}}-\frac{{\left(\mu_{g}-\sigma_{g}-\mu_{i}^{*}\right)}^{3}}{3\sigma_{i}^{*2}}+\frac{{\left(\mu_{i}+\sigma_{i}-\mu_{g}^{*}\right)}^{3}}{3\sigma_{g}^{*2}}-\frac{{\left(\mu_{i}-\sigma_{i}-\mu_{g}^{*}\right)}^{3}}{3\sigma_{g}^{*2}}.$$

Assuming equilibrium, set arbitrarily at $\mu_{g}^{*}=0$ and $\sigma_{g}^{*}=1$, I obtain two terms:

(9) $$\left[\frac{{\left(\sigma_{g}-\mu_{i}^{*}\right)}^{3}}{3\sigma_{i}^{*2}}+\frac{{\left(\sigma_{g}+\mu_{i}^{*}\right)}^{3}}{3\sigma_{i}^{*2}}\right]+\left[\frac{{\left(\mu_{i}+\sigma_{i}\right)}^{3}}{3}-\frac{{\left(\mu_{i}-\sigma_{i}\right)}^{3}}{3}\right].$$

Now, I will assume at equilibrium that $\sigma_{i}^{*}=\sigma_{i}$ and that $\mu_{i}^{*}=\mu_{i}=1.0$. I deliberately chooose $\mu_{i}^{*}\neq \mu_{g}^{*}$ because each individual is not necessarily at the group mean and I select unity arbitrarily. Holding all other parameters fixed (so $\mu_{g}=\mu_{g}^{*}$ and $\mu_{i}=\mu_{i}^{*}$), Equation (10) results.

(10) $$\left[\frac{1}{\sigma_{i}^{2}}\right]+\left[\frac{{\left(1+\sigma_{i}\right)}^{3}}{3}-\frac{{\left(1-\sigma_{i}\right)}^{3}}{3}\right]=\left[\frac{1}{\sigma_{i}^{2}}\right]+\left[2\sigma_{i}+\frac{2\sigma_{i}^{3}}{3}\right].$$

Equation (10) is plotted as a function of $\sigma_{i}$ in Figure 1 (black solid line). Observe that the two terms work in opposite directions, leading to a minimum shown as a ★ in Figure 1. The first term is the negative log probability (free energy) that the group will align with the agent at fixed $\theta_{i}^{*}$, which will be lower (more probable, lower free energy) if the agent is more “flexible” (can show a face the group will like, blue line in Figure 1). The second is the negative log probability the agent will align with the group, which will be lower if the agent is more precisely defined (i.e., more “findable,” red line in Figure 1). Although in this case it is simply because I assumed we were at equilibrium, it will in general be true because the individuals are more homogeneous.

There are numerous assumptions and shortcuts in the above analysis, but my objective was to derive a first approximation to the free energy of a group. The assumption that group and agent are fixed are quite restrictive, and the analysis above simplifies the simultaneous change of agent to group and from group to agent by using the symmetry of the problem. This simplification allows me to hold one agent fixed and modulate the other (or hold the group fixed and modulate the agent). Nevertheless, any more complex and reciprocal change would be characterised by the same equations, except with perhaps a coordinate change. Thus, I have proceeded with loss of generality only in the assumptions made (such as $D_{o}=⌀$), but relaxing these assumptions would generate multiple interesting avenues for future work. Using non-Gaussian distributions may be informative.

Generality is also reduced by the fact that I left out external forces altogether. However, such forces could be added to the equations above, and would share responsibility for D (along with the agent and group). Adding such elements may skew the overall structures shown in Figure 1, but will not change the core ideas I am presenting. This does, however, remain for a topic of future research.

### 2.2. Bigger or Smaller Groups

Now I remind you that the first term in Equations (9) and (10) in fact represents the entire group of N−1 individuals. Therefore, by weighting the two terms equally (black line in Figure 1), I have made an implicit assumption that the group is fully connected, so that there are N−1 terms like the first in Equations (9) and (10), that is, the individual interacts with everyone. This is not likely to be the case, however. What is more likely is that the newcomer interacts with only a dozen colleagues and managers, so his influence on the group is small. If we approximate this linearly and weight the first term in Equation (10) arbitrarily by N=100, then the dashed curves in Figure 1 result. The optimal configuration of parameters for free energy minimizing agents has shifted *rightwards*, and more individual flexibility is called for in order to integrate the individual into the group. Note that there is an arbitrary scaling: N = 1 means the arbitrary scaling factor being applied to the group/individual trade-off.

Nevertheless, the individual may have to change more than the group, as the weight of the population is in their favour (he is outnumbered). However, if the individual’s parameters are substantially mis-aligned in general with the group’s but aligned with some sub-group’s parameters, then if the social network is constructed in such a way that this individual is mostly interacting with the sub-group, then these models may be strengthened within the sub-group. Should the group become large enough, or socially organised enough, their skepticism may be able *”to offer a challenge to the upholders of the ‘official’ tradition”* ([12], p. 121). This challenge may be handled by merger into the main institution (*internalization* by the sub-group of the primary group), which then enriches and differentiates this tradition, or by segregation of the skeptics, a process of *objectivation* that possibly includes dehumanization (change of their agreed upon assigned identity). Finally, the sub-group may gain sufficient strength to form a political party and trigger change, in which case the existing traditions are thrown away and replaced with the new ideology, and the sub-group *externalises* to the group, society is produced by this sub-group, who define the new reality [12]. The definition and recognition of official sub-groups may be able to steer this process from an institutional perspective (see Section 3.1).

### 2.3. Flashlight Allegory

**Figure 3:**
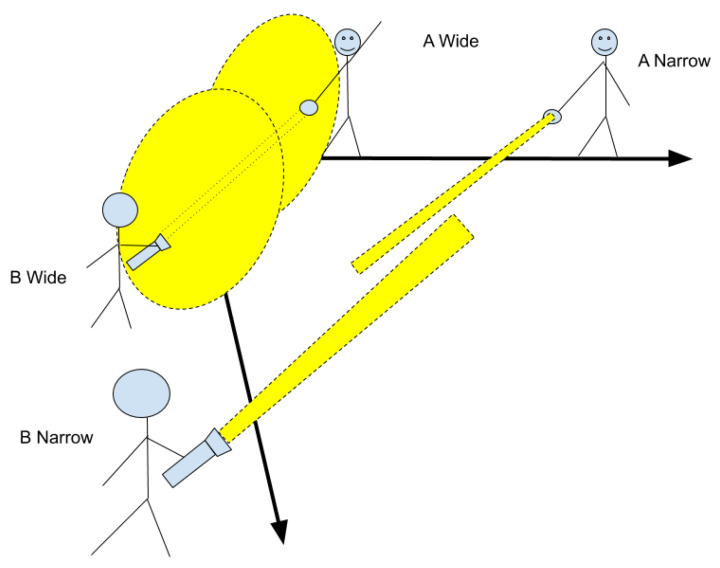
Allegorical example to demonstrate the uncertainty matching principle. Boy A (he can be A-narrow or A-wide) and boy B (B-narrow or B-wide) attempt to find each other’s flashlights and are rewarded by the density of light falling on the other boy’s flashlight.

I will present this balance problem using an allegory of two boys searching for each other in the dark with a flashlights, as shown in Figure 3. The flashlights have an adjustable beam width, from narrow and far to wide and close. The boys get rewarded for how much light the other records, or the **density** of light falling on him. One can see that for certain settings of flashlight beams, the boys have no hope. If one sets his beam on small and far, but the other does as well, they will have trouble finding each other. If both beams are wide, they can easily find each other, but the density of both together is low. Thus, they can either both use medium beams, or one can use a small beam and the other a large one.

In the flashlight allegory above, consider the targets for each boy (the other boy) are like the social world, and the flashlight is the boy’s predictions of how the social world will behave on a level of “meaning.” Thus, I am treating a group of agents as a single agent here, to simplify the presentation. The size of the target is the diversity of the social world, and represents the variance in expected behaviours. The size of the flashlight is the strength of the abstract social model the agent is building (his prior model). Therefore, the “allowable” settings are those that combine high diversity with strong abstract predictions and those that combine low diversity with weak abstract predictions. These settings may both work well in a network of agents with the same settings, but this does not mean that the agents are homogeneous. While their parameter settings may be the same, the parameter settings define the *space* of possible models and agent can take on, and are more of a measure of a social group’s expansiveness. Granted, boys with wider flashlight beams will have settings that rely less on abstract meanings. Also, individuals may conflict when put in groups with different settings, as the existing models will necessarily break down (and not match). The process of learning the new “fit” will be one that may be individual dependent. Such “spotlight” metaphors have been deeply explored in the context of psychological (usually visual) attention [16].

If we also take into account how many connections link up the group members (the density of the network, or the effective group for any agent), then the group component becomes dominant and larger, it being harder for the whole group to shift towards the agent (blue dashed line in Figure 1), and the resulting free energy has a sweet spot that has shifted *rightwards*, towards more freedom, shown in Figure 1 with ∘. Such a shift may also be caused by the *intensity* of the relationship to the group. Those relations formed in primary socialization, for example, may have much more intensity, and therefore a much bigger effect, than those formed in secondary socialization ([12], p. 152).

Work on latent structure learning of groups has shown that the assignment of a person to a group can be highly context dependent, as well as being dependent on dyadic similarity. That is, if Agent A meets Agent B, then how agent A categorizes agent B is dependent on how similar B is to each of A’s prototypes of groups or identities (e.g., a “doctor” is an identity, part of the group “doctors,” which is part of the group “medical professionals,” etc.), but also whether or not agent C is present, and their similarity and group behaviour with B [17]. If group similarity is higher, then the group becomes more fixed in its relationship with the new agent, and the agent is more likely to assign other agents to the new group than to some other, more loosely defined group. That is, the larger, tighter groups will have more “gravity” pulling people towards them. Such groups are mobilizations of people into political parties, institutions embedded in the social fabric of the group and capable of swaying public policy.

Finally, the flashlight allegory ties back to the division of epistemic labour mentioned in the introduction. If we replace one of the boys with a group of boys, then we can see the value in this matching process. Each boy is simultaneously playing the same game, alone, trying to “match” the other N−1 boys. The places where their flashlights meet (the rewards they receive for playing the game properly) are in the innovations illuminated by their crossing beams. As the location from which the beam emanates in some degree represents which particular boy is standing there, the bigger the group, the broader the range of beams. A broader beam is easier for the group to find, but harder to make bright enough to solve the problem.

### 2.4. Two-Level Models

The one-dimensional analysis in the previous sections is somewhat simplistic, but I can generalize to a two-level model fairly easily, which is what this section will discuss. Generalizing beyond two levels, or beyond one group, requires further study. Throughout this section, do not lose sight of the fact that all probability distributions I refer to define the relative likelihoods of state *and of actions/behaviours*.

Multilevel systems are interesting because they are both neurologically plausible and information theoretically rich. Each level in such a model has a certain degree of uncertainty to it, where uncertainty is really a characterization of degree of belief, following a Bayesian view that puts the existentialism of the world into the mind itself [18]. Further, there must be uncertainty in the *connection* between the levels, which turns out to be important. That is, once we propose two different functional “levels” of processing in the brain, they must be combined in some way to produce, in the end, motor signals for purposeful action. The way this combination happens can be more or less precise, that is, the levels depend more or less on each other. I will call the three types of uncertainty denotative (objective model), connotative (subjective model), and connective (objective-subjective connection model). There is no constraint on what the model actually *is*, so long as it has a use for these three types of uncertainty. Further, since there are actually approximately five levels in the brain, I would expect at least nine types of uncertainty. I focus here on three primary ones as exemplary, where denotative corresponds roughly to language, while connotative to social emotions or sentiments.

This type of “dual-process” model is known to have parallels in human brain function and behaviour. However, it considers the role of abstract (some of it emotional) reasoning as a group-level process, and that of deliberative thought as an individual one. This is contrary to many modern views of deliberation and rationality as a group process (e.g., the “rational” economy), while emotion is individual and causes irrational behaviour. I make a distinction between *action* and *behaviour* in that the first describes linguistic labels (propositions) denoting actions, such as *give something to*, while the second describes the affective meanings of an action, say very positive and a bit powerful in this case.

Although these ideas generalize to other models, if using a probabilistic, two-level model, the state of the top level can be viewed as representing the *parameters* of the predictive model the next level down. Observations are then represented by the state at this next level down, and its dynamics are represented by the state one level up. Inference in this model is both state estimation and learning of the parameters of the low-level model, and is the definition of Bayesian machine learning. In what follows, I consider a particular type of two-level Bayesian model in which the “high” level is a continuous state parameter which is taken a priori to be the dynamics of sentiment as measured in population surveys, and the “low” level represents the dynamics of the objective, outside world. Such a model is restrictive, but gives me an easier way to relate to models of political freedom in Section 3.

### 2.5. Bayesact

*BayesAct* is a two-level model of human intelligence and affective reasoning (individual and social) that explicitly represents the three types of uncertainty in a simple and measurable way by leveraging the machinery of affect control theory (ACT) [9,19,20,21,22]. ACT is a model of emotional coherence based on language that was founded on the control principle of Powers [23], which states something very reminiscent of the free energy principle: that people try to minimise incongruencies by controlling their perceptions. Heise transposed this to the sentiment space of Osgood et al. [24], imposed a denotative structure from symbolic interactionism [25], and added affective dynamics [26]. ACT is a computational model that has been used to predict classes of human behaviour in a variety of settings [27]. ACT maintains a deterministic and static denotative model as an actor-behaviour-object-setting state (e.g., “doctor advises patient in clinic”), and an associated deterministic, but dynamic, connotative model. This connotative model is a a dynamical system in Osgood’s three-dimensional space of affective meaning: evaluation, potency and activity. This dynamical system represents *values*, or *evaluative knowledge*, which can be contrasted with *declarative* and *procedural* knowledge that are represented in the denotative model.

*BayesAct* combines these mechanics with a formal decision theoretic model, a partially observable Markov decision process, or POMDP [28,29], extensively used in operations research [30]. A POMDP instantiates a *temporal frame* or *structural representation* [31]. Frames, as schemas, are a classic structure used in early artificial intelligence (AI), Knowledge Discovery and Data Mining (KDD), and Information Retrieval (IR) research that assigns a label and interpretation to each object, fact, relation and event that constitute a particular situation. Such structures are typically logical and discrete-valued to enable ease of use in a computer program. For example, we might label the positions of pieces on a chess board, or predictions about how a game will turn out given a sequence of moves, or the bids in a negotiation. The inclusion of the connotative meanings of ACT means the model must be augmented with labels for identities and behaviours corresponding to ACT’s denotative model, but with added noise modelling. These labels can then be interpreted as distributions in a sentiment space using a measured dictionary. This sentiment space thus complements the *denotative* state I have been describing so far, with a *connotative* state (which in fact is 18 dimensional). The model is fundamentally based on the symbolic interactionist idea that symbols (language) provide order for “the subjective apprehension of biographical experience” ([12], p. 97). Symbols are then reified elements of exactly these same subjective apprehensions.

Thus, learning and being become one single experience. The combination of symbolic and affective interpretations is what enables *generalization*: once the symbol “doctor” is assigned to someone, expectations for her behaviour become defined as generally as possible with respect to her occupation. That is, I expect her to do something good and powerful, but I am open to a range of actual objective actions that could be in play in the current situation. For example, if the current situation is a court-room, I still expect her to do something good, such as testify honestly, and powerful, such as speak authoritatively. If she is coaching my son’s hockey team, I also expect her to be honest, fair and caring. If my son’s hockey coach is a policewoman, I may expect a more authoritarian and disciplinarian experience for my son. Note that both my assumptions may be wrong as this individual may be enacting a completely different identity while coaching.

Frames form the foundation of much knowledge representation work in AI, but have been efficiently implemented using Bayesian networks (BNs), which can be used to compute a distribution over all possible worlds modeled by a particular frame [18]. This probabilistic model then rests on the structural ontology and temporal logics that are proposed in the frame. Bayesian decision networks generalise the *goals* in frames as preference functions that rank all possible outcomes using a numeric scale, e.g., a utility function [32]. *BayesAct* complements this denotative model (the variance of which is called *invalidity*), with the ACT-based connotative model (the variance of which is called *coherence*), and a model of the relationship between them, the *somatic transform* (the variance of which is called *dependence*) [6,33]. For example, in a government policy decision, the facts may include the amount of money spent or saved, and long-term estimates from potentially complex predictive models, and the utility is financial or based on some index of social well-being. The denotative temporal dynamics may describe immediate and longer-term effects, enabled by adding more latent state, and allow for the construction of a policy that optimises over some definition of utility based on the same features. The denotative temporal dynamics may also encode *norms of behaviour* that indicate the normative choices to make for any given identity-behaviour combination (e.g., a “citizen” should not “free ride” on other “citizens”).

The connotative dynamics are ACT-based, and will encode the relative freedom trade-offs for whatever group they are applied to. That is, for some particular configuration of the denotative state, including a definition of identities, a connotative distribution results that may be used to compute how emotionally coherent various behaviours are. This connotative coherence is one of “feeling” or “intuition,” which may override any norms. I will call such coherence “prescriptive” rather than “normative.” A striking example is a trolley problem, in which it is logical to throw the switch on a runaway trolley so that it kills only one person instead of five, a strong connotative prescription against “killing someone” may take over for many people and prevent this logical strategy. Another example is an ultimatum game, in which one player is given $10, and can give any amount he wishes to the other player. While logically the proper amount to give is $0 (or 1¢ if the game is repeated and the other player has a choice not to play), most humans will fork over approximately 20% to 40% of the amount they are given, with the amount being culturally dependent [34].

### 2.6. Three Types of Uncertainty

The two-level model discussed in the last section has three sets of parameters governing denotative, connective, and connotative elements. The three parameters are denoted δ, γ, and α, respectively. I therefore project the overall freedom-equality dimension from Figure 1 into a three-dimensional space.

**Figure 4:**
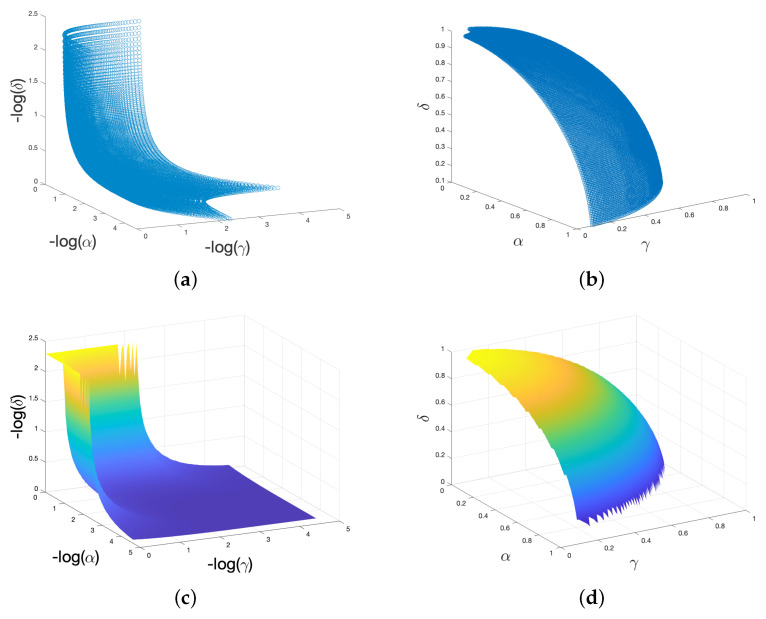
Views of the minimum free energy points along each ray from the origin. (**a**,**c**): Axes are −log(parameters), but correspond in scale to coherence ($-\log(\hat{f}f)$), dependence (−log(γ)), and validity (−log(δ)). I plotted the negative logarithm of each parameter, and colored the surface in (**c**), for visibility only. (**b**,**d**): Axes are the raw variance parameters (so larger is more dispersed, more freedom).

Equation (9) is the free energy for a one-dimensional parameter space, under certain assumptions. In a three-dimensional space, we can imagine this free energy curve, as shown in Figure 1, varies along any ray emanating from the origin, and that the minimum point defines the surface of the “simplex,” which is therefore revealed to be more of a “dome” shape (assuming radial symmetry). I therefore plot the simplex by seeking the minimum free energy along each ray from the origin. Plotting this as a function of $-\log\theta \propto \theta^{-1}$) yields Figure 4a, with an interpolated, smoothed version in (c). Figure 4b,d are the same plotting θ directly.

**Figure 5:**
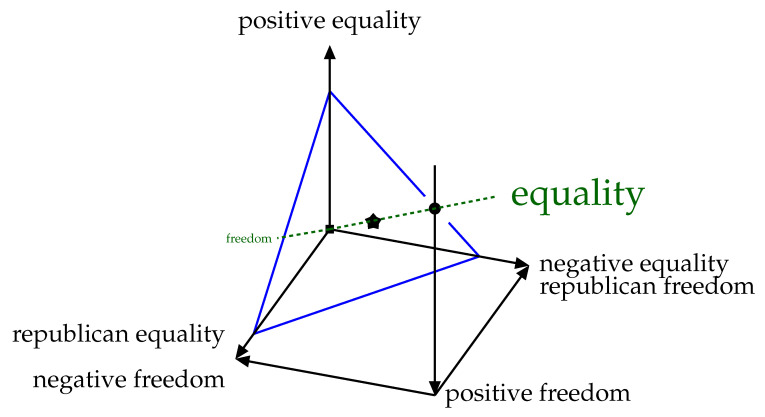
Simplex on the three dimensions of freedom and equality. Also shown are poles of perfect freedom *■* and perfect equality •, The star is in the most central position possible for a social agreement on the management of uncertainty.

Since free energy increases with an increase in any parameter of the three, in order to be at equilibrium, it must decrease in at least one of the other two. What this implies is that the three-dimensional parameter space is in fact a two-dimensional surface of equilibrum, at each point of which the free energy is at a minimum. I have imposed a restriction here by assuming the decrease is the same; however, there may be some arbitrary scaling that may arise due to the physical nature of our environment. I make a radial assumption in Figure 4, which presents the information in three dimensions with as little added bias as possible (simply what this theoretical model is telling us). However, because of the assumed arbitrary (relative) scaling of parameter sets in the *BayesAct* model, viewing this surface as a simplex as in Figure 5 is easier to relate to theorizing about human freedom and equality, as in Section 3. The exact shape of this surface may not be as shown in Figure 4 or Figure 5, but recall that a social system becomes increasingly difficult to arrange as you move out along any dimension of freedom, and thus the actual range of operation of these parameters is likely to be relatively small, centered around a region in center of the minimum free energy manifold.

## 3. Freedom in Social Groups

While the model in the last section boils things down to three complementary sets of parameters, the non-determinism in social groups may be substantially more complex. However, as I will show in this section, they can also be boiled down to three complementary sets of parameters. First, consider what we mean by uncertainty. Often, non-determinism can be reduced to an estimate of how likely some outcome is to occur, given some policy of action: this is the *risk*. Risk is an important concept, because if one can define risk, and one has fixed preferences, then one can make a decision-theoretically optimal decision about behaviours that lead to this risky outcome. That is, an agent can rationally decide whether or not to do something, and be right about it, only when the risk is something she can estimate. However, if she cannot estimate the risk (perhaps she has never tried the behaviour so has no statistics to learn from about the likelihood of the outcomes), then her estimate of risk itself is uncertain, and we label this type of “meta-” uncertainty as “ambiguity,” or the “unknown unknown” [35]. The reason ambiguity is important as a separate concept, is that it is a factor determining when people rely more on social than individual learning, alongside problem difficulty and learning cost ([36], p. 64).

There are two main reasons why an agent would no longer be able to estimate risk properly. The first is there may be some unknown (to the agent) factors that influence the outcome. These factors might be discovered should the agent try the behaviour, which it cannot do reliably without an estimate of risk. The second is the agent may lose the capacity to model an environment that has become too complex. There is a third reason risk may be hard to estimate, which is essentially the same as the first: the cost of a behaviour may be too high. This implies the agent cannot do the action, and so leaves the outcomes unknown as in the first reason for ambiguity. These three reasons are both known to be important in gauging if an agent will favour social learning (learning by imitating others, for example) over individual learning (e.g., learning by evaluating outcomes decision theoretically) ([36], p. 64). In either case, I will call the resulting environment *invalid* [37], which is synonymous with *ambiguous*, but less ambiguous.k

### 3.1. Three Freedoms

Ambiguity is handled by people in three complementary ways, which correspond to three things at play: the group, the individual, and the connection between the individual and the group. Another way of saying this is the objective (external, the group) the subjective (internal, the self) and the connective (membership in the group). The representations of the social context in an agent’s brain or mind pervades reason and thought, and the way in which each agent in each context trades off the social and individual contexts will be defined by, and will define, the social order and thus reality: *“the relationship between the individual and the objective social world is like an ongoing balancing act”* ([12], p. 134). Therefore, these three locusses of ambiguity management lead to three concepts of freedom, *Republican*, *Positive*, and *Negative* which I now explore using the framework of Anderson [38].

Republican freedom means people are not subject to anyone’s unaccountable will, and is also known as *independence*. As republican equality is increased, then everyone becomes equivalent and dependent. Normally this is done by making all dependent on a sovereign or a monarch, such that all independence is removed by subjugation to the monarch’s unaccountable will. However, a smart and honest monarch gives his subjects lots of opportunities (positive freedoms) and lets them have free choices (negative freedoms) but can intervene at any time to impose an arbitrary will to ensure everyone is steering in the same direction.

Positive freedom implies opportunity, implemented by slackening constraints at the group level, meaning uncertainty must be managed at levels lower down (individual) and higher up (at the corporate or government level). Positive equality means that opportunity is more constrained. Positive equality is a place where everyone is exactly acting in the same way and the world is predictable and *valid* [37]. Therefore, if you could maximise positive equality, then everyone would act according to a single plan. One such plan could be a rational plan. By defining what is good and what is bad, a rational decision maker can be used to set policy. This definition also equates to the ontologies used to classify people and groups, as those considered “bad”, e.g., those labeled “madmen and children” ([39], p. 33) can be excluded in order to preserve rationality. The power to make this definition may be abused by a despot for personal gain.

Negative freedom is defined by the freedom an agent has to choose its own actions, from whatever choices it is given. So moving towards negative equality means removing people’s abilities to choose their own actions. One way to do this is by defining affective identities, and then making more stringent requirements on how actions should be coherent with these identities, as explored in Section 2.4. These culturally approved dynamics become institutionalised, and they remove negative freedom of individuals to act in whatever way their will directs them. Thus, an increase in emotional coherence between (seemingly self-imposed) actions and behaviours, in an emotionally stratified society, leads to a reduction in the space of actions under consideration, accompanied by a corresponding increase in negative equality in which actions are constrained by social prescriptions. A state of “world closedness,” extracted from a state of “world openness,” is a result ([12], p. 51). The classic imposition on negative freedom is private property. I can wall off a piece of ground for myself, and I have increased my negative freedom on my property. Although I still require the law, and an enforcement component of government to ensure this freedom is upheld, I have decreased the negative freedom of 7 billion people (realistically, only a few hundred co-citizens of my rural town), and therefore overall have increased negative equality.

Negative and positive freedom can be easily confused. The important difference is in *where* this freedom lies. Positive freedom is a property of the group of agents. The more open the group is to new ideas, for example, the more positive freedom it affords its members. Negative freedom is a property of the individual. As individuals are mostly constrained by the presence of others, negative freedom is decreased when positive freedom is increased through diversity, for example. Although I have the positive freedom in my country to stand outside and shout my opinions, I do not have the negative freedom to do that as I would be ashamed that my neighbors may see me. As more diverse preferences surround me, there are more of such things that will reduce my negative freedom further.

### 3.2. Social Capital

Defining “social capital” as the emotional bonds in a network of people [40], I find that it can be implemented in two ways. First, by restricting republican freedom but allowing negative and positive freedoms, one gets a tight-knit group of homogeneous, intolerant individuals devoted to the group. Such a group is rich in “bonding social capital” and have low tolerance, e.g., a “sectarian community” ([40], p. 355). Second, by restricting negative freedom but allowing positive and republican freedoms, one gets a highly diverse and tolerant group, but one that must be trusting of others. Such a group is rich in “bridging social capital” and has high tolerance for out-group members, e.g., a “civic community” ([40], p. 355). Putnam [40] also discusses two other forms of societies, those with high tolerance but low social capital (of either sort) are “individualistic” (every man for himself), and those with low tolerance and low social capital (“anarchic”). While the individualistic case implies no positive freedom but complete negative and republican freedom, the anarchic case implies complete freedom across the board, and is not workable as a societal solution given even natural diversity due to statistical fluctuations. Fukuyama has also written extensively on the idea of *trust* [41,42], which he equates with *social capital* [40] and *cultural values* ([41], p. 110).

### 3.3. Ternary “Simplex”

Anderson [38] presents these three freedoms as both *distinct* (in that they can be individually varied) and valuable (in that all are worth something). There is evidence that they vary inversely with respect to each other (e.g., gains in republican freedom are usually traded off against losses in negative freedom in a social democracy). If we make one assumption that an increase in one such freedom means an increase in overall freedom, then a group at an equilibrium of trading off freedom and equality would tend to increase equality in response, to restore equilibrium. What dimension is increased would not matter, but all cannot be increased (or decreased) at once. Thus, these three freedoms form a ternary structure (in which only one can be maximal at a time), and so I postulate three freedom-equality dimensions as shown in Figure 5, and so that it appears as a dashed green line in Figure 5. Freedoms increase down each axis towards the freedom pole at the origin (■) in Figure 5. Each type of equality (freedom) is increased by moving away from (towards) the origin along the corresponding axis.

## 4. Discussion

Politics, from the laws themselves to the people who make them, enforce them, and evaluate them, are based on some degree of balance between *freedom* and *equality*. As ([43], p. 96) points out, *“every system of law [has] two main objects, freedom and equality.”* However, there are many ways to balance these two elements. For example, one group may value everyone’s freedom to act (e.g., to carry a gun), while another may value everyone’s equality of action. One can easily see that one cannot be free and be equal in a society of others. If everyone is free, then there will be inequality. If everyone is equal, then no one is free. In the words of  ([44], p. 171): *“The liberty of some must depend on the restraint of others.”* Freedom and equality are heavily discussed in the literature, of which I will barely skim the surface. My primary objective with this paper is to show that the different kinds of freedoms enjoyed by people are related in a non-trivial way to some information theoretic principles about the management of uncertainty.

I can represent this definition of freedom and equality on a single axis, as shown in Figure 1. On this figure, a society could be set up anywhere along the line between freedom and equality. However, using free energy principles on a one-dimensional model, I can show that there is a “sweet spot” at which the group functions most efficiently. This sweet spot, shown in Figure 1 with a ★, is a minimum of free energy for the group, and is defined by how uncertainty is managed in a group of agents (see Section 2). The natural equilibrium of the group is when the group and its members are in harmony. Another way to say this is that any group attempting to settle *away from* this sweet spot, will be less efficient, and may be dominated by groups who are at their own sweet spot. Learning where an agent should situate its own, internal model of the world is something non-self-interested that an agent does, but it is something that benefits the group as a whole. Due to a host of exogenous factors the group will be unlikely to be found at their “sweet spot,” but rather would look like a small cloud in the three-dimensional space, with more density somewhere along this simplex. To get a sense just how much variation is found in such a cloud, one can consider how to implement collective intelligence through rewards, as in [45], but seeing collective intelligence as a property of the group, not of the individual, leads to a different interpretation in which the group prescription is the norm, and the individual’s rational deliberations lead it astray.

I therefore conjecture that freedom is an estimate of the capacity of posterior belief distributions in a hierarchical model which includes agent policies. Different types of freedom express themselves at different levels. Similarly, I define equality as the inverse of this: an estimate of the precision of the same posterior belief distributions. Very precise distributions require people to be less diverse, more similar, more equal. This conjecture allows me to connect Figure 5, derived from social theory, to Figure 4, which is derived here analytically from information theoretic principles, but could potentially be derived from data by building artifacts that actually fit into and become members of a social group.

As a simple example, consider the diversity of a population. We can represent diversity as a distribution over a range of human attributes, plotted along the *x*-axis in Figure 1 as model capacity. Higher capacity allows a wider range of attributes, leading to a more diverse population with a lot of freedom. With reduced capacity (to the left) in Figure 1, comes reduced diversity, so people are spread across a smaller number of attributes, everybody is very much the same, and there is much more equality.

Those operating in the society of diversity are more often going to run into diverse views of things, and therefore they will learn a more uncertain or “spread out” view of their society. They will therefore be more free to choose their own actions as there will be less constraint from the group level (as it is more spread out). Agents that live in the homogeneous society are going to have very precise distributions over the other agents in their group since everyone is similar. Actions are constrained, but equality and security are guaranteed. Security is guaranteed because, if everyone is the same as you, then you can be very certain about things, you are in a state of pure equality, and you get pure security as a reward: you can predict what is going to happen next. If everyone is very different, then you will be very uncertain about how people will act but you can be free to act in any way you want because it will not stand out and people will know how to handle it.

Degrees of uncertainty are therefore intimately connected with freedom. What types of freedom are associated with each of these three type of uncertainty? Denotative uncertainty is the same as positive freedom, in that invalid environments are ones in which everyone is doing different things, and so positive freedom is maximised. When all are “forced” to behave in some way (e.g., rational), then positive equality is maximised (everyone is following the same plan), but of course, positive freedom is minimised.

Connotative uncertainty is the same as negative freedom, as it releases people from social norms and prescriptions that cause constraints on their actions. Note that I am making this association primarily on the basis of using ACT as a model for the connotative state. Seen from a strictly Bayesian model selection viewpoint, the connotative state is the *family* of models that are being used to make predictions about the effects of action on the world. Those using the same family of models, say Gaussian processes, will be solving problems represented the same way (same *perspectives* [8]). They may be using the same heuristics as well to solve their problems, at which point generating diverse solutions will be difficult, and negative freedom is reduced. They may also be using different heuristics, which gives them an advantage by allowing them to divide labour and act cooperatively. Those working from different model families (different perspectives) will find synchronization more difficult. However, they may also gain advantage from their diversity due to the “diversity trumps ability theorem” [46], which leads to two conclusions: “Diverse perspectives are more likely to lead to breakthroughs and to create communication problems. Diverse heuristics are more likely to lead to smaller, more iterative improvements” ([8], p. 239). In the first, putting together different models leads to an increase in negative freedom, whereas in the second, shared model families lead to a decrease in negative freedom, but may increase positive freedom.

Institutions, as a family of models, increase negative equality. An institution has an organizational “culture” that increases social norms and prescriptions, and decreases negative freedom. In my analysis above, I assume everything is in equilibrium, so that all model families are the same. However, there is much to be gained from studying how this system behaves out of equilibrium.

Finally, connective uncertainty is the same as republican freedom, as it releases people from adherence to some externally defined reference point. A great deal of connective certainty requires a leader, who, since positive and negative freedoms are maximised in this state, must be authoritarian. This leader must define what is “good” and “bad” for the group to be cooperative, since everyone within the group has so much freedom to follow their own definitions. Connotative certainty also requires a leader, but it can be defined as a social contract, since this configuration requires people to give up their negative freedoms to obtain this connotative security.

Therefore, the setting of parameters of uncertainty (variances) in a two-level Bayesian model of each agent corresponds to the setting of political belief in the resulting group and the placement on a three-dimensional simplex of freedoms. The primary insight is that all such settings are equivalent in terms of their trade-offs between equality and freedom *in general*, that is, along the dashed green axis in Figure 5, which is what we really should care about. The precise way in which this balance is achieved matters less, and conflicting mixtures of uncertainty management should be avoided. Narratives that can give justifications for actions in line with one group or the other may be important to guide marginalised groups towards fair solutions. Losses of republican freedom can be compensated for by underlining the associated gains in positive freedom, for example.

As with the single-dimensional (linear) version in Section 2, there are numerous different ways of achieving the same freedom-equality trade-off. The trade-off shown in Figure 1 happens along all three freedom axes. In order to handle this, I assumed that the trade-off in Figure 1 operates along any radial vector in this three-dimensional space. Variations on this assumption may yield different results. The simplex gives us a convenient way to discuss the manifold shown in Figure 4, so long as we remember that in practice it has this particular scaling. Note that the minimum free energy goes to *∞* as any parameter drops to zero, as Equation (9) blows up. It is therefore more difficult to plot the simplex as a function of the inverses of the parameters (Figure 4a,b). Regardless of how we talk about this space we have to end up on (or near to) this two-dimensional surface shown outlined in blue in Figure 5. This surface is a *simplex*, and represents the “sweet surface” of free energy. It may not actually be a plane, but rather a spherical shape or bowl shape, see Section 2.6, but this planar approximation sweeps arbitrary scaling under the rug and gives us a useful analytical tool.

The three freedoms that I have been describing can be related to the three different ways of managing uncertainty in a two-level Bayesian model. More generally, I believe these same three trade-offs in uncertainty will be happening across all levels of the brain, and may be generalizable using the approach of Gilead et al. [47], in which each level abstracts (is the “abstractrum”) from the level below (the complementary “concretum”, which itself may be an “abstractum” of a further level). Such a hierarchy would vastly increase the modelling capacity of each agent, and thus of the group. The parameter space would, however, would have more dimensions, and so focussing on only two levels and three dimensions may give us insights in the construction of such a more complex model, while maintaining some explanatory validity.

## 5. Conclusions

In this paper, I have presented a highly abstracted model of a group optimization of free energy. I have shown how, under certain assumptions, a group of agents jointly minimizing free energy can be represented by a set of agents who learn from each other. Such agents will tend towards models with similar levels of dispersion, which is related to how much capacity for modelling the outside world they have. I further discuss a Bayesian hierarchical model with a hybrid state space that I relate to sociological theorizing about small group behaviours. I show how a trade-off in dispersion or capacity is present across the levels in this model, and discuss how these trade-offs relate to common notions of freedom in societies.

The model I am presenting is necessarily simplistic, and does not come close to approaching the complete gamut of tools and techniques used by humans to coordinate behaviour. In fact, one could view an entire society as a "cloud" of small distributions in this three-dimensional space, forming one large distribution. A cloud that is constantly moving as situations change and agents interact with one another and learn. A society can also likely be pulled forcefully into one or another configurations; however, their natural tendencies might operate clandestinely to provide a countervailing force.

Nevertheless, I find it compelling that the properties of the parameter space of this hierarchical Bayesian model seem to reflect some of the properties of people’s understanding of freedom and uncertainty. This upwards reduction, if carried to its logical extreme, leads to a somewhat different philosophical view that denies primacy of individual states, or at least accords social states with equal status. In this view, everything is situational, although part of the situation is the agent itself, including all its strategies, planning, and decision making. However, these are not considered individual traits at all, but rather social constructs that are learned and applied in a given situation. In this view, “personality” is just a bag of tricks that a group has learned, and are not some inherent property of any given person. This philosophical view denies the primacy and stability of “personality” as a fixed and stable trait.

Individuals both try to make sense of the world they are in, and try to define it. They are faced, however, with an information asymmetry (principal agent problem), in which they cannot even represent, let alone understand, the complexity of their social groups. Thus, individuals, as principals, are forced to offload some of that computation onto other agents (as agents of the principal). The more they do this, the more similar to those other agents they become, and the more homogeneous the society becomes. However, if they do less of it, they become more independent, which the group favours as it leads to flexibility, the ability to handle the unforeseen (the “Black Swans” [48]) and the ability to assimilate new members. The derivation I have presented in this paper puts *learning* of “preferences” (as predictive distributions) as central to the collective decision making process, and does not assume individuals share predictive models (are all rational), violating two basic assumptions of economic theory [49]. While the analysis was simplistic, any number of the assumptions made could be lifted (such as radial symmetry) in order to see if and where the connection breaks down. Using normally distributed models in *BayesAct* is restrictive to allow for analysis, but I believe that using other distributions (e.g., with broader tails) would yield similar results, and the three-way trade-off would still show through.

A number of directions are currently being pursued, mostly directed at explaining a variety of so-called *heuristics and biases* in terms of this one unifying model. An initial paper shows how dissonance and fairness may be related to socio-emotional reasoning [50]. Work on confirmation and narrative biases is ongoing. Confirmation bias may function similarly to narrative bias in that both have sharpened denotative models as a result of further evidence (more other people opting for it, or more precise statements), and so these tend to be rated as more likely. Non-normal probability models are also under consideration.

## Data Availability

Information about and supporting data for *BayesAct* can be found at bayesact.ca.
